# On verbal working memory. Descriptive study in post-intensive care syndrome patients after COVID-19 infection in a functional rehabilitation unit in Spain. A pilot study

**DOI:** 10.1192/j.eurpsy.2021.1764

**Published:** 2021-08-13

**Authors:** M.J. Maldonado-Belmonte, E. Fernández-Jiménez, M.T. Sánchez-Polo

**Affiliations:** 1 Clinical Psychology, Hospital Central de la Cruz Roja, Madrid, Spain; 2 Idipaz, Department Of Psychiatry, Clinical Psychology And Mental Health, La Paz University Hospital, Madrid, Spain; 3 Rehabilitation, Hospital Central de la Cruz Roja, Madrid, Spain

**Keywords:** Verbal working memory, COVID-19, Post-Intensive Care Syndrome, Clinical Neuropsychology

## Abstract

**Introduction:**

Post-Intensive Care Syndrome (PICS) is a physical, cognitive, emotional and functional condition resulting from prolonged stays in ICU (Intensive Care Unit). In pathologies with clinical characteristics similar to SARS-CoV-2 pneumonia, most patients showed cognitive deficits after discharge from ICU. Further studies are needed on verbal working memory among PICS patients.

**Objectives:**

To analyse the verbal working-memory performance among patients with PICS after COVID-19 infection in a Functional Rehabilitation Unit in Madrid (Spain) using the Spanish version of the Screen for Cognitive Impairment in Psychiatry (SCIP-S).

**Methods:**

This study was conducted in the Hospital Central de la Cruz Roja, in Madrid (Spain). A sample of 17 PICS adult patients was included, with age ranging from 56 to 74 years old (mean = 68.35 years; 13 males). Patients were assessed around three weeks after referral from their reference hospital. The Working Memory Test (WMT) of the SCIP-S was used as outcome. Descriptive analyses were conducted (mean and standard deviation) on standardized scores (z) based on age-adjusted general population norms. Significant impairment was set at z < -1.5.

**Results:**

Mean z-score on WMT was -.64 (S.D. = .60) from the total sample, with 5.9% of cases with significant impairment (mean = -1.53).

**Conclusions:**

These preliminary results show low probable presence of impairment on verbal working memory among PICS patients after COVID-19 infection. Longitudinal studies, with larger samples, are needed where the premorbid cognitive level is considered.

**Disclosure:**

No significant relationships.

